# Vancomycin dosing in patients undergoing maintenance hemodialysis

**DOI:** 10.1007/s11255-014-0707-0

**Published:** 2014-04-03

**Authors:** Aleksandra Rymarz, Dorota Brodowska-Kania, Małgorzata Gomółka, Elżbieta Józefczak-Bergier, Małgorzata Dzierżanowska, Stanisław Niemczyk

**Affiliations:** 1 Department of Internal Diseases, Nephrology and Dialysis, Military Institute of Medicine, Szaserów 128, 04-141 Warsaw, Poland; 2 Department of Laboratory Diagnostics, Military Institute of Medicine, ul. Szaserów 128, 04-141 Warsaw, Poland

Editor,

In recent years, increasing pathogen resistance for vancomycin has been observed. According to new recommendations, trough target levels for this drug should be between 15 and 20 µg/ml [1]. Concentrations lower than 10 µg/ml can cause therapeutic failure and bring about vancomycin resistance [2]. To achieve this target, an initial dose of 20–25 mg/kg actual body weight is recommended for all patients.

We examined 22 patients (5 female, 17 male), age 64.32 ± 12.2 years, being treated with hemodialysis for end-stage renal disease between 2011 and 2012. The study protocol was approved by the Ethics Committee of Military Institute of Medicine. All patients were treated using a low-flux dialyzer of varying sizes three times a week for 3–4 h. Vascular accesses were as follows: cuffed catheters (16 patients), noncuffed catheters (five patients) and arteriovenous fistula (one patient). Each patient received an initial dose of vancomycin 20 mg/kg actual dry body weight, rounded by 250 mg with an infusion rate 10 mg/min after dialysis session. Vancomycin trough levels were determined twice: 4 days after the initial dose and 4 days after the second dose. The second dose was related to plasma vancomycin concentration. If vancomycin concentration was at target level (15–20 µg/ml), the same dose was repeated. If concentration was lower than 15 µg/ml, the second dose was increased by 30 %. If vancomycin concentration exceeded 20 µg/ml, a reduced dose was administered. Fluorescence polarization immunoassay test (Axsym system, Abbott) was used to assess plasma vancomycin concentration.

The main reason for vancomycin administration was sepsis (20 patients, 90.9 %), in all cases related to catheter. The most frequent pathogen was methicillin resistant Staphylococcus aureus (13 patients, 72.2 %).

Mean trough vancomycin concentration after the initial dose was 13.26 ± 4.46 µg/ml. Mean vancomycin dose per actual body weight was 20.62 ± 2.35 mg/kg. Mean first total dose was 1,590.91 ± 342.98 mg. Six patients (27.2 %) achieved target trough levels of 15–20 µg/ml. Range 10–20 µg/ml achieved 72.7 % (16 patients). 15 patients (68.1 %) had trough levels below 15 µg/ml, among them six patients (27.2 %) had levels below 10 µg/ml. Only one patient had concentration above 20 (21.42 µg/ml).

After the second dose, the mean trough level of this drug was 20.73 ± 4.58 µg/ml. Mean vancomycin dose per actual body weight was 23.01 ± 10.11 mg/kg. Mean total second dose was 1,777.78 ± 351.85 mg. Nine patients (40.9 %) had trough levels of 15–20 µg/ml. Two patients had trough levels below 15 µg/ml, and no one fell below 10 µg/ml. Eleven patients (50 %) had concentration above 20 µg/ml, among them mean concentration was 23.42 µg/ml.

Initial vancomycin doses in hemodialysis patients should be based on actual body weight; however, one-third of patients do not reach the recommended trough level of 15–20 µg/ml. It seems reasonable that among certain patients higher doses could be used. Subsequent doses of the drug should be related to its serum concentration, type of membrane used in dialysis session and time of its administration. Vancomycin dosing protocol in hemodialysis patients requires further evaluation on larger groups of patients.
